# Restoration of 1325 teeth with partial-coverage crowns manufactured from high noble metal alloys: a retrospective case series 18.8 years after prosthetic delivery

**DOI:** 10.1007/s00784-021-04063-8

**Published:** 2021-07-09

**Authors:** Philipp Rehm, Hermann Derks, Wilfried Lesaar, Benedikt Christopher Spies, Florian Beuer, Mats Wernfried Heinrich Böse

**Affiliations:** 1 Private Dental Office Dr. Rehm, Klückenhofstraße 1, 46459 Rees, Germany; 2 Department of Prosthodontics, Geriatric Dentistry and Craniomandibular Disorders, Charité – Universitätsmedizin Berlin, Corporate Member of Freie Universität Berlin, Humboldt-Universität Zu Berlin, Aßmannshauser Str. 4-6, Berlin, Germany; 3 Private Dental Office Dr. Hermann Derks, Steinstraße 12, 46446 Emmerich am Rhein, Germany; 4 Center for Dental Medicine, Department of Prosthetic Dentistry, Faculty of Medicine, Medical Center – University of Freiburg, University of Freiburg, Hugstetter Str. 55, 79106 Freiburg, Germany

**Keywords:** Dental crowns, Partial-coverage crowns, High noble metal alloy, Prosthodontics, Dental materials, Tooth preservation, Gold alloy

## Abstract

**Objectives:**

To evaluate long-term survival and success rates of conventionally cemented partial-coverage crowns (PCCs) manufactured from high noble metal alloys (hn).

**Material and methods:**

Restoration-, periodontal- and tooth-related criteria on patients, restored with a single or multiple conventionally cemented hnPCCs in a private dental office were collected from existing patient records. With regard to semi-annual follow-ups, data of the most recent clinical evaluations were considered. Kaplan–Meier and log-rank tests were used for statistical analyses. Level of significance was set at p ≤ .05.

**Results:**

Between 09/1983 and 09/2009, 1325 hnPCCs were conventionally cemented on 1325 teeth in 266 patients (mean age: 44.5 ± 10.7 years). Due to various reasons, 81 hnPCCs showed complications, documenting a success rate of 93.9% after a mean observation period of 18.8 ± 5.7 years. Of these, additional 14 restorations were counted as survival, resulting in a survival rate of 94.9%. Most frequent complications were periodontal issues (*n* = 29, 35.8%). Significantly higher success rates were documented for hnPCCs of patients aged between 37 and 51 years (*p* = .012).

**Conclusion:**

Partial-coverage crowns from high noble metal alloys showed excellent survival and success rates after a mean observation period of 18.8 ± 5.7 years. Higher patient age was one of the risk factors.

**Clinical Relevance:**

According to the results of this study, hnPCCs still represent an excellent therapeutic option—even in modern dentistry.

## Introduction

Dental structure can be damaged by various reasons and requires different conditional treatments to ensure the preservation of the remaining tooth substance. Defects can be restored in a direct and indirect approach. Limitations for both procedures exist, but a recommendation of when to use direct and indirect restorations is not clearly described in the literature [1]. However, for large defects with cusp involvement or complicated residual occlusal surfaces, mainly indirect techniques are used.

Indirect restorations have various advantages especially when used for defects that affect the stability of teeth and contain one or more cusps. Thereby, partial-coverage crowns (PCCs) are restorations for lost tooth structure, requiring a minimally invasive and technically challenging procedure, while preserving more residual tooth substance compared to complete crowns (CCs). They are superior to direct restorations with regard to approximal and occlusal contacts, surface quality and marginal fit [2, 3]. Due to changing demands regarding precision, stability and esthetics, new materials with different characteristics are constantly introduced on the dental market. In general, metal alloys, dental ceramics and composite materials can be differentiated. Metal-based and metallic-colored restorations have been declining in recent years, because the desire for tooth-colored restorations has become more important in society. Despite the possibility of veneering metallic materials, they cannot match the esthetics of all-ceramic restorations and therefore became increasingly unpopular. The slightly allergenic potential of metal alloys (in particular the contained palladium) and the expense considering high noble metal alloys compared to dental ceramics should also be considered [4]. Additionally, the smooth surface and the low surface tension result in less plaque retention on dental ceramics compared to metal surfaces or enamel [5].

Nevertheless, ceramic restorations also have disadvantages compared to hnPCCs. The most frequent failures of these materials are veneering porcelain fractures or fractures in general [6–8]. Furthermore, a retentive preparation form is not mandatory for ceramic partial-coverage crowns (cPCCs), but in return, the complication-prone adhesive bonding is limited to situations with adequate moisture control. With regard to the variety of available composite resin cements, allergies were reported associated with these materials [9]. Results regarding marginal gaps vary enormously depending on the type of ceramics and the process of manufacturing whereas the marginal gaps for hnPCCs are documented at a low level in the literature [10–12].

High noble metal alloy restorations have been used for over 140 years to restore compromised teeth [13]. It should be critically evaluated whether ceramic restorations can compete with the clinical performance (“gold standard”) of hnPCCs [14]. Material characteristics such as a minimal risk of fracture, the advantages of ductility and the associated adaptation to the occlusal concept that changes over time (contact wear of loaded teeth) of hnPCCs were described in the literature [15–17]. Due to the ductility of gold, hnPCCs can adapt to different antagonistic materials like enamel and direct or indirect restorations of all kinds [18].

The long-term preservation of teeth and especially the preservation of masticatory function is one of the major goals in dentistry. Forecasts on the survival rate of partial crowns vary between 70 and 96% after 5 to 11 years in the literature [19, 20]. The heterogeneous results are often related to short observation periods [20, 21] and differences regarding the treatment procedure in terms of preparation technique, impressions, choice of material and laboratory procedures [19].

The aim of this retrospective case series was to investigate long-term survival and success rates of hnPCCs with already collected and available clinical data. It was intended to demonstrate the possibilities of a consistent clinical protocol and its strict implementation using materials long established. The working hypothesis is that hnPCCs continue their eligibility as an option for restorations of decayed teeth in dentistry with good long-term prognosis.

## Material and methods

### Study design and ethical request

The present study was designed as a clinical retrospective case series and ethical approval was given by the Ethical Committee of Charité – Universitätsmedizin Berlin, Germany (application number: EA4/135/19). All treatments, subsequent inspections and data collection were conducted in the private dental office of and by the authors H. D. and W. L. under a constant protocol.

Anonymized data provided were retrospectively analyzed and statistically evaluated. Patient inclusion criteria were as follows: (1) Treated with at least one hnPCC between 09/1983 and 09/2009 in the private dental office of the author H. D.; (2) Attended at least twice a year for annual examination; (3) According to necessity, regular professional teeth cleanings or supportive periodontitis therapy has been implemented. In total, 1325 hnPCCs met the aforementioned inclusion criteria and were considered in this study.

### Course of treatment

**Fig. 1:**
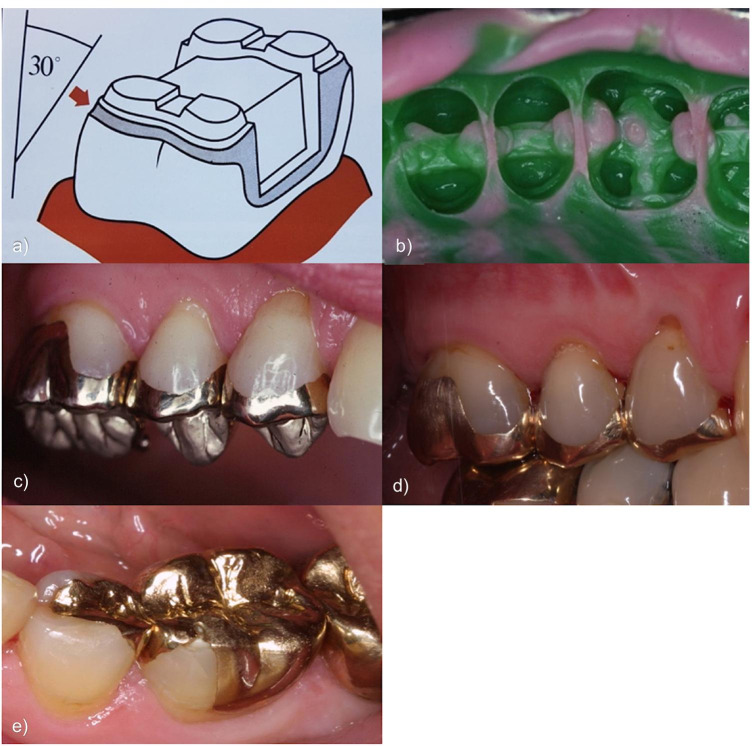
Exemplary pictures regarding preparation design, impression, and restorations with hnPCCs. **a** Preparation design with a bevel of 30° to minimize the cement gap [22, 23], **b** impression with hydrocolloid, **c** “buttons” for easier removal after fitting, **d** hnPCCs in situ after 30 years of service and **e** hnPCCs not covering all cusps causing increased risk of prismatic fracture [19, 24]

Following necessary and successful pretreatments, all teeth preparations were conducted according to the guidelines of Schulz-Bongert [22]. Ideally, the load-bearing cusps were reduced by 1.2–1.5 mm and the shear cusps by 1.0 mm. Occlusal boxes were preferably prepared with a depth of 2 mm, a width of 1.5 mm and a taper of 3–10 degrees. Both proximal contacts were dissolved and likewise prepared with a taper of 3–10 degrees. Non-functional cusps and the preparation margins were chamfered (Fig. 1a). A substance removal of at least 0.8 mm was required to meet the minimal thickness of the restoration material.

The subsequent impressions were made with a hydrocolloid impression material (Aqualoid Green, The Belport Company, Camarillo, CA, USA) and hydrocolloid impression trays (DC Hydrocolloid Impression Tray, DentalCentral GmbH, Hamburg, Germany, Fig. 1b). Impressions of the opposing jaws were made with alginate (Alginat rosa regular set, Omnident Prisman GmbH, Lorsch, Germany) and Rim-Lock impression trays (Rim-Lock Impression Tray, Dentsply DeTrey GmbH, Konstanz, Germany). An additional hydrocolloid impression was taken to fabricate an unsawn control model. Through the years, three different face bows and corresponding articulators were used (Whip mix, Whip Mix Corp., Louisville, USA; SAM, SAM® Präzisionstechnik GmbH, Gauting, Germany; Artex CR, Amann Girrbach AG, Pforzheim, Germany). Bite registrations were performed in habitual occlusion with wax plates (Integra Miltex, IntegraLifeSciences Service France, Saint-Priest, France) and a temporary cement (TempBond NE, KerrHawe SA, Bioggio, Switzerland). The Dawson handle was used to take the bite in centric position in case several restorations were performed with loss of the support zones and/or a present temporomandibular disorder (TMD).

Lab models were fabricated from type IV plaster (Implant Rock, picodent, Wipperfürth, Germany) and mounted (pico-arti new edition, picodent, Wipperfürth, Germany) in the aforementioned articulators. The hnPCCs were conventionally modelled with wax (S-U-Ästhetikwachs, SchulerDental, Ulm, Germany) and embedded (gypsum-bonded investment, easysoft picodent, Wipperfürth, Germany). All restorations were cast from a high noble metal alloy (Degulor M, DentsplySirona, Bensheim, Germany) and fitted to the models. To simplify try-ins, the restorations were provided with small buttons (Fig. 1c). If necessary, final adjustments (i.e. correction of occlusion) were performed on matted surfaces. All hnPCCs were conventionally cemented with zinc phosphate cement (Harvard, fast setting, Richter and Hoffmann, Berlin, Germany). Finally, static and dynamic occlusions were checked with 12-µ-thick occlusion foil (HANEL occlusion foil, black and red, Coltène/Whaledent AG, Altstätten, Switzerland).

### Study parameters

The regular clinical examinations included an update of the medical history, and dental and periodontal parameters. Success of each of the included hnPCCs in this study was determined considering (1) restoration-, (2) periodontal- and (3) tooth-related criteria [25].

The evaluation of restoration-related parameters included different aspects comparable to the modified United States Public Health Service criteria (USPHS modified by Cvar and Ryge) (Table 1) [26]. Initially, for a rating as success, examined hnPCCs had to be in situ and undamaged [25]. In order to verify that, tactile examination of the surfaces, marginal gaps and possibly presence of secondary caries was performed with a double-ended dental probe (EX9, HuFriedy, Chicago, USA). Visible or palpable perforations of hnPCCs led to an exclusion from a rating as success.

**Table 1:** Individually adapted criteria for clinical evaluation of inlays and onlays, using the modified United States Public Health Service criteria according to Ryge and Cvar (1971), compiled by S. P. Studer et al. [26]

1) Marginal adaption	
A	Margin not discernible, probe does not catch, no discoloration visible
B	Probe catches on onlay margin but no gap; gap or chipping on probing, with enamel exposed, but polishable; slight discoloration visible, but polishable
C	Gap or chipping with dentin or liner exposed; distinct discoloration visible, not polishable, not acceptable, secondary caries
D	Partial fracture, fracture, luxation or mobile (loose) restoration, fracture of abutment tooth
2) Anatomic form	
A	Correct contour with tight proximal contacts (checked with waxed dental floss) (exception: diastema situation), no wear facets on restoration, no wear facets on opposing teeth
B	Slightly under- or over-contoured, weak proximal contact; small wear facets on restoration, diameter ≤ 2 mm; and/or same on opposing teeth
C	Distinct under- or over contoured, missing proximal contact; large wear facets on restoration, diameter ≤ 2 mm; and/or same on opposing teeth
3) Surface texture	
A	Smooth, glazed, or glossy surface
B	Slightly rough or dull surface
C	Surface with deep pores, rough, or unevenly distributed pits, cannot be refinished

The used criteria related to the USPHS were categorized as follows: (1) marginal adaption, (2) anatomic form and (3) surface texture. Each of the criteria was assessed as A, B, C or (D), where A was the highest and C/D the lowest rating. Restorations with an A rating were considered as success, whereas restorations with minor defects, not endangering the tooth structure, pulpal or periodontal tissues and could be renewed, reattached due to loosening, fixed in any way (i.e. polished) or received further invasively therapies were rated as B and therefore as survival.

Ratings with C or D were counted as failure. Abutment teeth with primary caries, endodontic treatment, periodontal treatment or extractions, and restoration used for other prosthodontic work were generally rated as C and therefore its restoration with hnPCCs as failure.

Circumferential periodontal pocket depths were measured (WHO probe, E. Hahnenkratt GmbH, Königsbach-Stein, Germany) at four sites per tooth (mesio-buccal, disto-buccal, disto-lingual, mesio-lingual) and classified healthy with pocket depths of up to 4 mm [27–29]. In addition, the degrees of loosening of the teeth were categorized according to the criteria of Miller [30] as follows: (0) “physiological mobility” measured at the crown level: the tooth is mobile within the alveolus approximately 0.1–0.2 mm in a horizontal direction; (I) increased mobility of the crown of the tooth: at most 1 mm in a horizontal direction; (II) visually increased mobility of the crown of the tooth: exceeding mobility of 1 mm in a horizontal direction; (III) severe mobility of the crown of the tooth: in both horizontal and vertical directions impinging on the function of the tooth [30]. Ratings as (0) and (I) were considered as success, whereby (II) was only considered as survival and (III) as failure.

For a rating as success, the vitality (Omnident Kältespray, Prisman GmbH, Lorsch, Germany) and percussion tests had to be inconspicuous. Vitality tests were performed with foam pellets and cold spray (Omnident Kältespray, Prisman GmbH, Lorsch, Germany). Teeth that were treated with a root canal treatment prior to prosthodontic treatment were excluded from vitality tests. Percussion tests were used to preclude apical inflammation and were performed in vertical and horizontal direction with a dental mouth mirror (MH1, HuFriedy, Chicago, USA). The assessment of both tests was documented by the author H. D. Hyper- and hyposensibilities, pain or a noticeable, altered sound led to further diagnostics.

Additionally, the following parameters were included in the statistical analysis: (1) gender (male, female), (2) age at delivery, (3) date of delivery, (4) location of hnPCCs, (5) jaw (maxilla, mandible), (6) supplied quadrant (I, II, III or IV), (7) total number of restorations and (8) date of follow-up. For statistical analysis, the patients were divided into four groups of equal size based on the number of cases and received restorations in order to obtain a strong statistical significance. In the first group, patients under 37 years of age were included, the second group included patients aged between 37 and 44 years, the third group patients aged between 44 and 51 years and the fourth group patients aged above 51 years. The classification is roughly based on DMS V [31], but could not be adopted exactly due to the uneven distribution of case numbers.

For a rating as a successful hnPCC, all criteria had to be rated as success themselves following the above-defined criteria.

### Statistical analysis

Including 1325 hnPCCs in 266 patients, data as gender, age, time of restoration in situ and localization were gathered for a descriptive analysis. The metric variables were presented as means and medians, while the scatter measures were presented as standard deviations and quartiles. The categorized or nominal data were given as absolute and relative frequencies. Success statistics were performed using Kaplan–Meier analyses. Continuous data were categorized by quartiles so that survival in the four groups could be compared (gender, age, location, quadrants and jaws, type of teeth). Log-rank test was used to compare the influence of co-variables (gender, age at loading with hnPCCs, location, quantity) regarding success probabilities. The significance level was set at p < 0.05. Statistical analyses were performed with SPSS (version 24.0, IBM, New York, USA) for Windows (Windows 10, Microsoft Corporation, Redmond, USA).

## Results

### Demographic data and survival

**Fig. 2:**
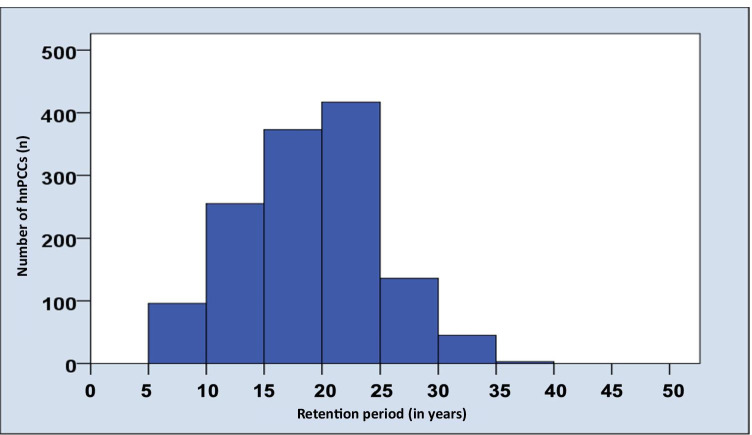
Bar chart representing the years of service of investigated hnPCCs

**Fig. 3:**
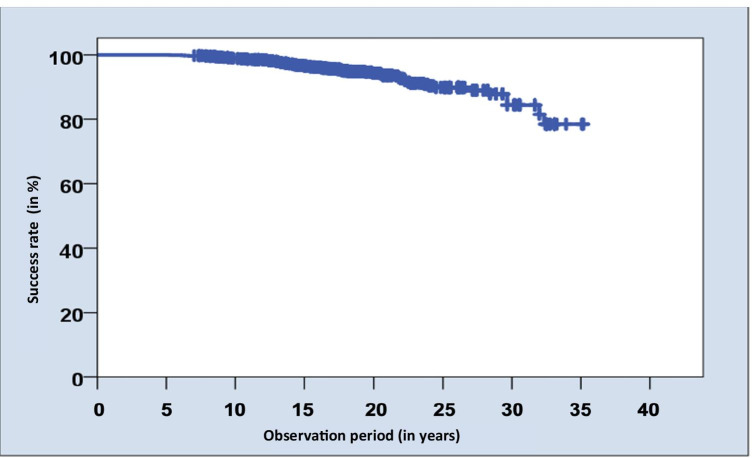
Kaplan–Meier survival analysis of hnPCCs up to 35 years. Censored subjects are indicated on the curve as tick marks

A total of 1325 hnPCCs were conventionally cemented between 09/1983 and 09/2009. In this study, previously anonymized data regarding patients with semi-annual follow-ups in a private dental office between 09/2016 and 03/2017 were evaluated (Table 2). One thousand two hundred and fifty-eight hnPCCs were still in situ at the time of this study after a mean observation period of 18.8 ± 5.7 years (Figs. 2 and 3), resulting in an overall survival rate of 94.9%.

**Table 2:** Influence of different variables on survival/success rates in absolute numbers and percentages

Variable	Total number of hnPCCs	Percentages	Success rates		
			Total number of successful hnPCCs	Percent %	
Gender					
Male (132)	692	52.2%	652	94.2%	
Female (134)	633	47.8%	592	93.5%	
Age groups					
< 37	335	25.3%	304	90.7%	
37 – 44	328	24.8%	313	95.4%	
44 – 51	330	24.9%	318	96.4%	
> 51	332	25.0%	309	93.1%	
Localization					
Canine	2	0.2%	2	100.0%	
1st premolar	85	6.4%	79	92.9%	
2nd premolar	161	12.2%	154	95.7%	
1st molar	421	31.8%	394	93.6%	
2nd molar	564	42.6%	530	94.0%	
3rd molar	92	6.9%	85	92.4%	
Jaw					
Maxilla	668	50.4%	630	94.3%	
Mandible	657	49.6%	614	93.5%	
Quadrants					
I	336	25.4%	312	92.9%	
II	332	25.1%	318	95.8%	
III	330	24.9%	307	93.0%	
IV	327	24.7%	307	93.9%	
Total	1325	-	1244	93.9%

All hnPCCs were conventionally cemented on teeth of 266 patients with a mean age of 44.5 ± 10.7 years at loading. Thirty-one patients were treated with a single hnPCCs (11.7%), 47 (17.7%) with 2, 37 (13.9%) with 3, 35 (13.2%) with 4, 24 (9%) with 5, 65 (24.4%) with 6–10, 26 (9.8%) with 11–15 and a single patient with 16 (0.4%). Six hundred and sixty-eight (50.4%) were cemented in the maxilla and 657 (49.6%) in the mandible (Table 2). Males were treated with 692 (52.2%) and females with 633 (47.8%) hnPCCs. The distribution of hnPCCs was as follows: 1077 molars (81.3%), 246 premolars (18.6%) and 2 canines (0.2%) were treated. Second molars were most frequently restored with hnPCCs (*n* = 564, 42.6%), followed by first molars (*n* = 421, 31.8%). Third molars (*n* = 92, 6.9%) were the least frequently restored molars. The distribution on premolars was as follows: 85 first premolars (6.4%) and 161 s premolars (12.2%). A canine tooth was restored twice (0.2%) in order to restore the canine guidance.

### Complications

**Fig. 4:**
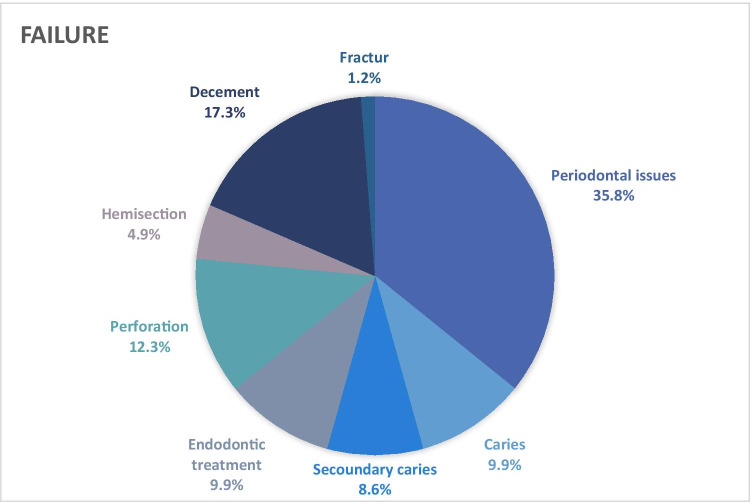
Pie chart regarding the different reasons for failure of investigated hnPCCs

Of 1325 hnPCCs and its supporting teeth, 81 (6.1%) showed complications after a mean period in function of 16.2 ± 6.2 years. Of these, 14 hnPCCs (17.3%) were re-cemented without additional efforts. These restorations were counted as survival but not as success. The most frequent complications were severe periodontal issues of *n* = 29 teeth (35.8%), which resulted in extractions. Other reasons for failure were perforations (12.3%, *n* = 10), caries (9.9%, *n* = 8), subsequently endodontic treatment (9.9%, *n* = 8), secondary caries (8.6%, *n* = 7), hemisection (4.9%, *n* = 4) or tooth fracture (1.2%, *n* = 1) (Fig. 4). Two of the decayed teeth and the fractured tooth had to be removed, resulting in the loss of 32 teeth (2.4%).

### Success rates and influencing factors

One thousand two hundred and forty-four hnPCCs met the defined requirements for success regarding the restoration-, periodontal- and tooth-related criteria. This resulted in an overall success rate of 93.9% after a mean observation period of 18.8 ± 5.7 years. The investigated parameters described in the “Material and methods” section showed different impacts regarding the individual success rates (Table 2).

**Fig. 5:**
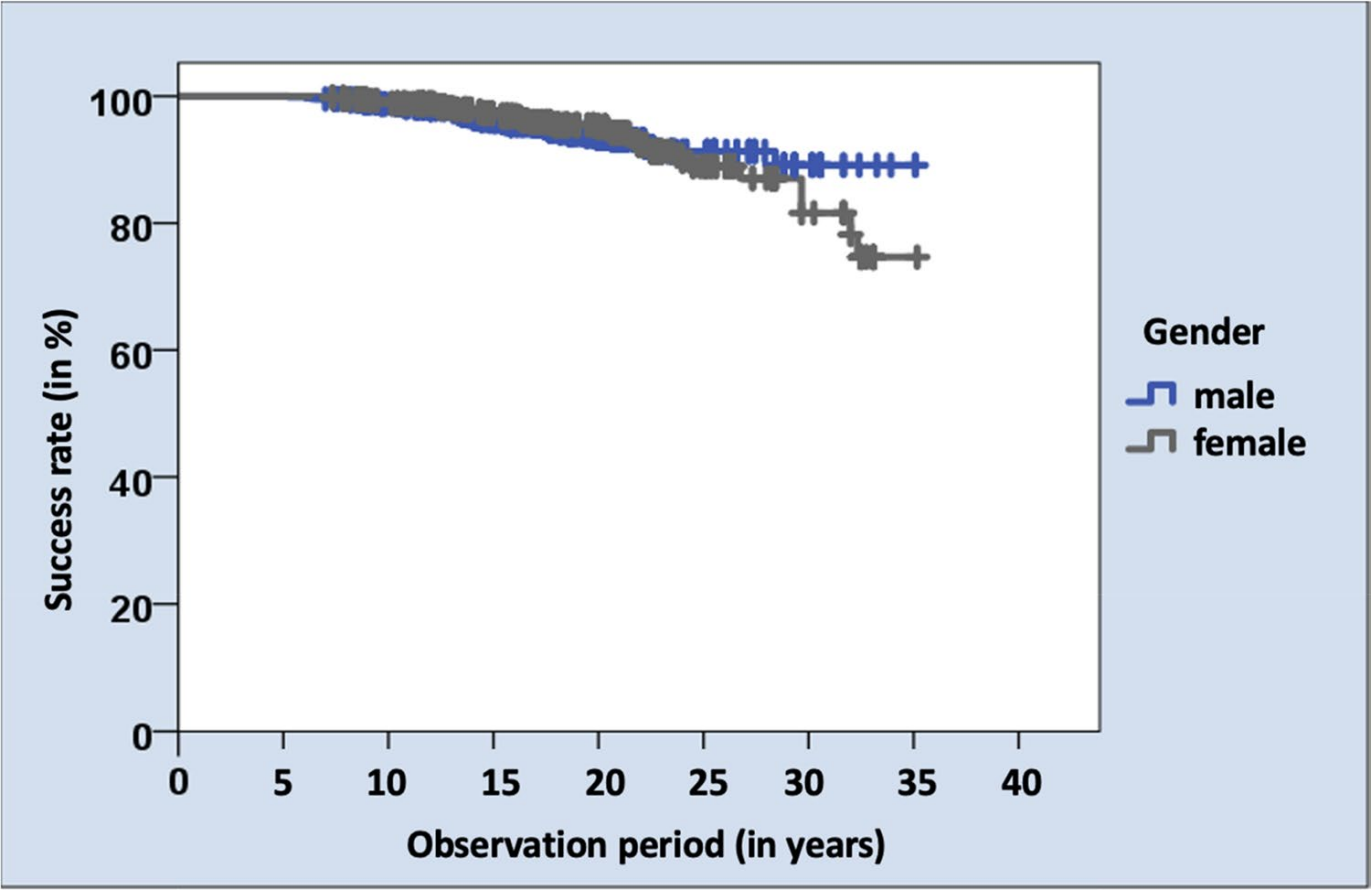
Kaplan–Meier survival analysis between gender and success rates of hnPCCs in percent in years

No statistically significant differences regarding its impact on success rates were documented for gender (*p* = 0.961). In men, 40 hnPCCs and in women 41 hnPCCs showed complications and therefore could not be rated as success. The documented success rates were 94.2% for men and 93.5% for women. Although after 30 years, the cumulated success rate in women decreased more strongly, it did not represent a statistical significant difference (Fig. 5).

**Fig. 6:**
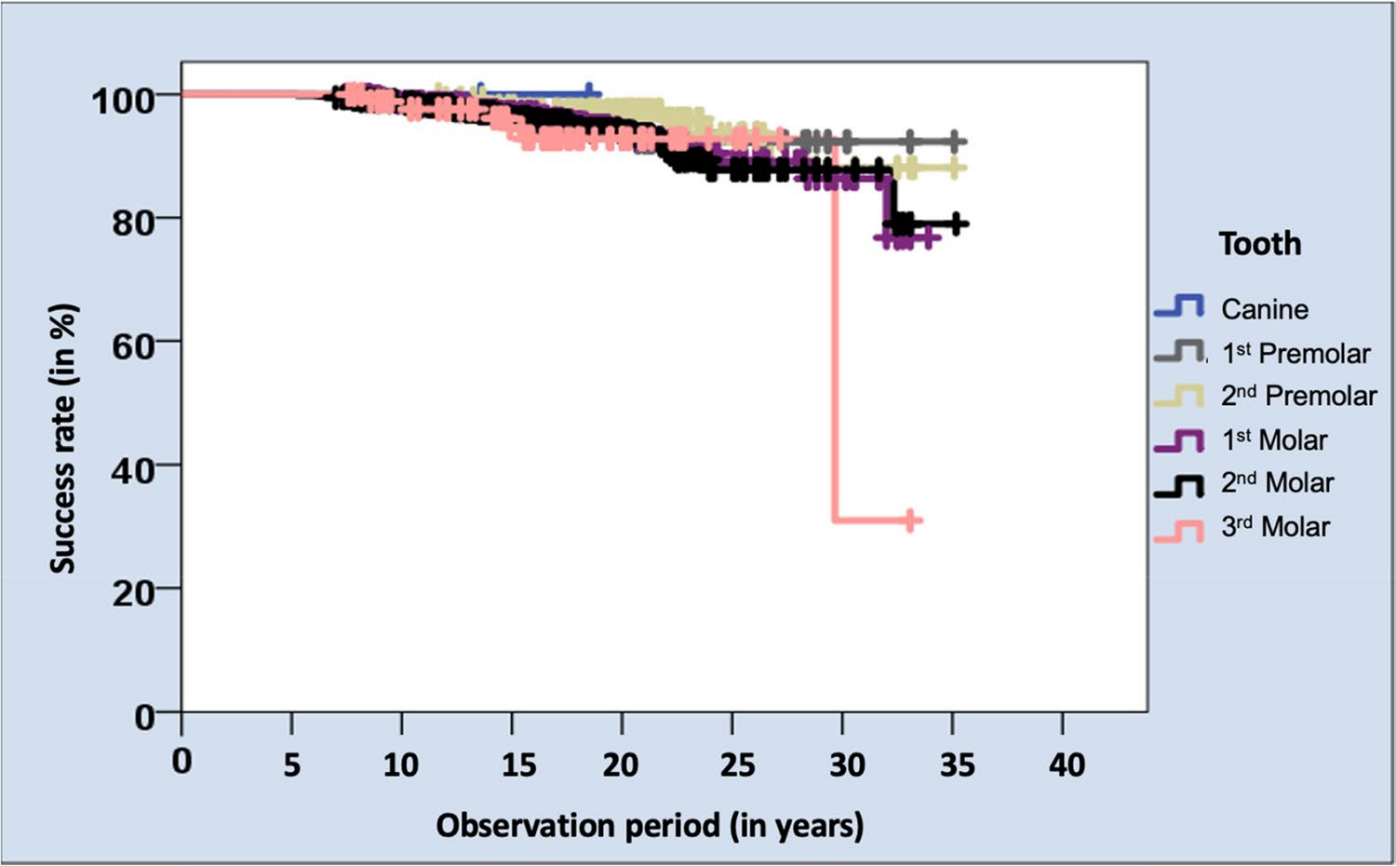
Kaplan–Meier survival analysis regarding the success rates of hnPCCs restoring respective teeth after an observation period of up to 35 years

The success rate for both the evaluated hnPCCs attached to canines was 100%, which, considered individually, could be neglected due to the small sample size. Second premolars showed the highest success rate with 95.7%, closely followed by second molars with 94.0%. First molars showed a success rate of 93.6%, first premolars of 92.9% and third molars of 92.4%. The different success rates did not represent a statistically significant difference (p = 0.475). However, again, the cumulated success rate, in this case for the third molars, decreased sharply after about 30 years of service (Fig. 6).

**Fig. 7:**
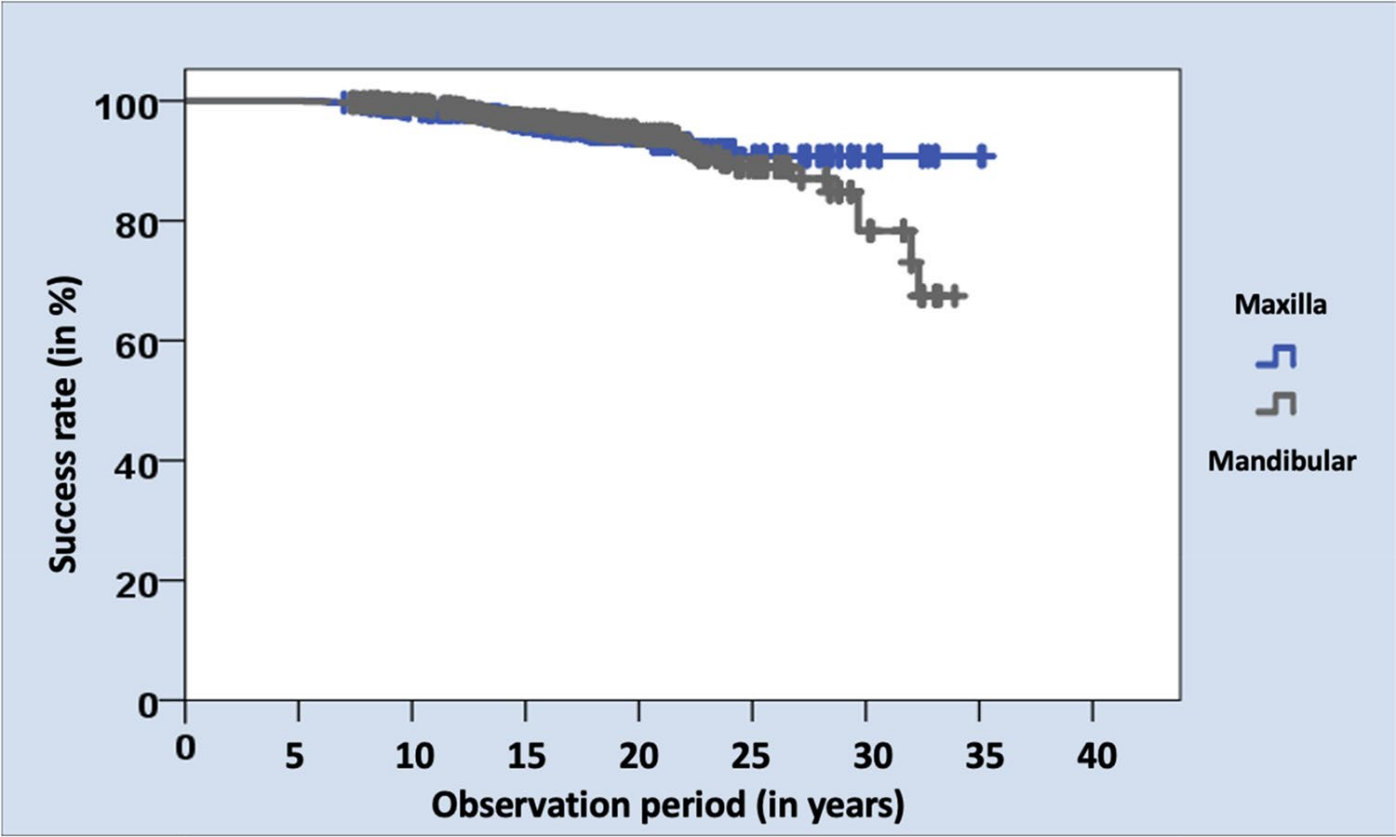
Kaplan–Meier survival analysis regarding the distribution of hnPCCs located in the mandible and maxilla

Furthermore, there was no significant difference in the success rates of hnPCCs in the upper jaw (*n* = 38 failures, success rate 94.3%) and the lower jaw (*n* = 43 failures, success rate 93.5%, p = 0.515, Fig. 7).

**Fig. 8:**
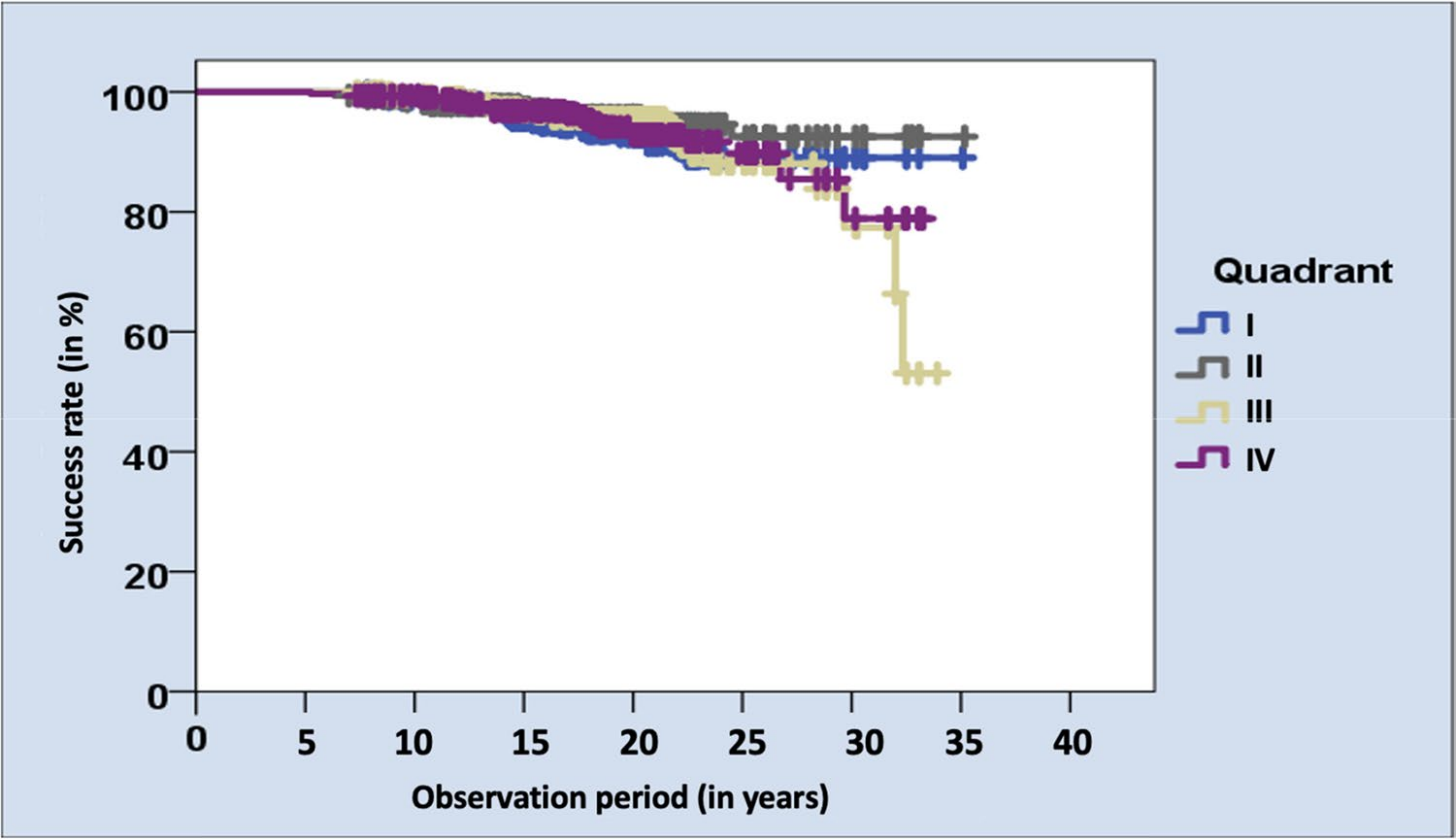
Kaplan–Meier survival analysis regarding the success rates of investigated hnPCCs regarding all four dental quadrants

The localization of attached hnPCCs regarding the four quadrants (I.–IV.) neither revealed a significant impact on the success rates (p = 0.435). In the first quadrant, *n* = 24 restorations showing complications (success rate 92.9%), in the second *n* = 14 (success rate 95.8%), in the third *n* = 23 (success rate 93.0%) and in the fourth *n* = 20 (success rate 93.9%, Fig. 8).

Kaplan–Meier survival analyses (Figs. 7 and 8) revealed that the success rates of hnPCCs in the lower jaw clearly decreased after an observation period of 30 years, compared to the other groups. However, no statistically significant difference could be calculated.

**Fig. 9:**
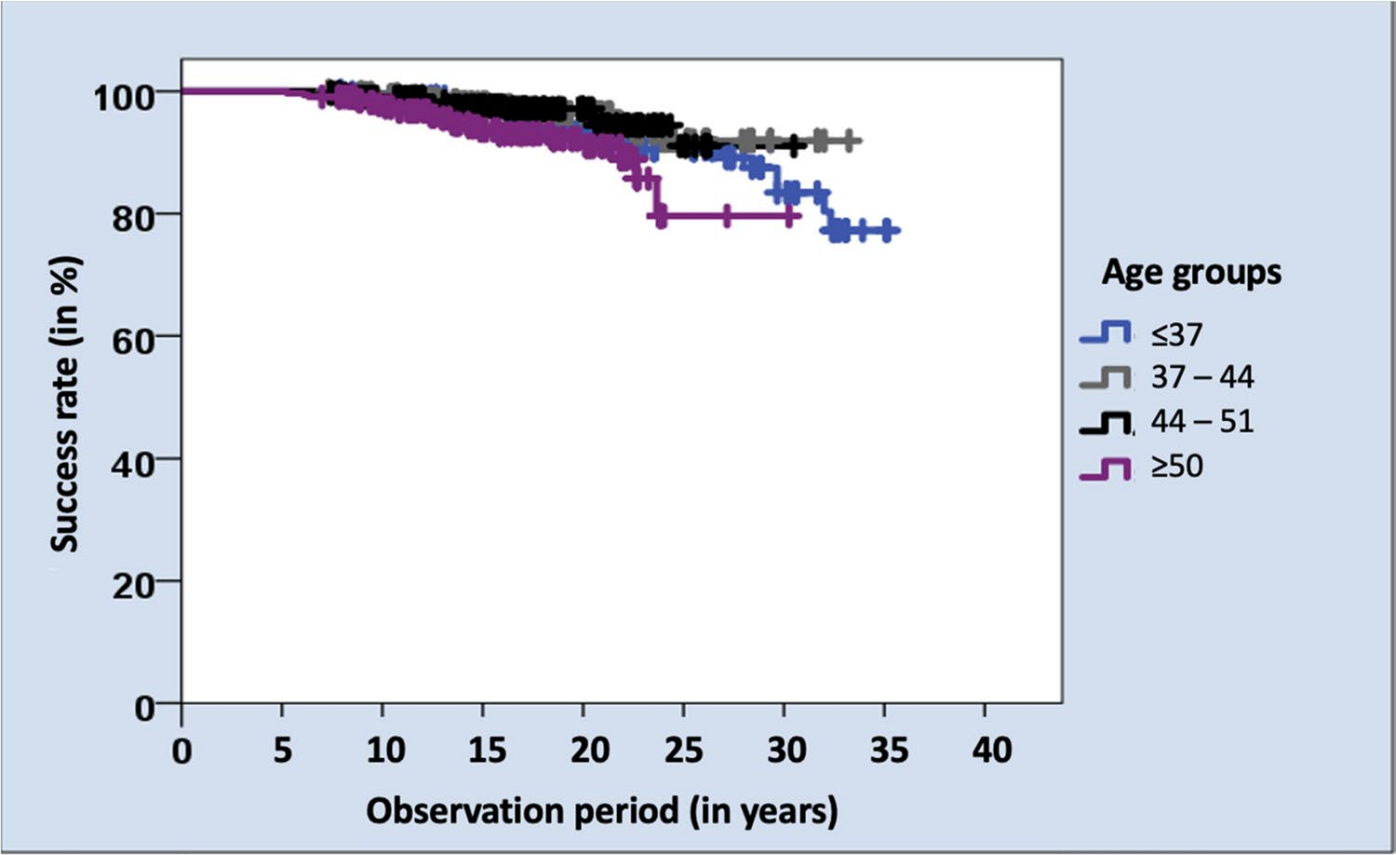
Kaplan–Meier survival analysis regarding the success rates of investigated hnPCCs regarding different age groups

The success rates of hnPCCs for the different age groups showed significant differences. High noble metal alloy PCCs of patients who were younger than 37 years (success rate 90.7%) and of patients older than 51 years (success rate 93.1%) revealed statistically significant (*p* = 0.012) worse success rates than the middle age groups (success rates 95.4% and 96.4%). Patients < 37 years showed complications in 31 cases, patients between 37 and 44 years complications in 15 cases, patients between 44 and 51 years complications in 12 cases and patients > 51 years complications in 23 cases (Table 2, Fig. 9).

Besides the overall success rate of 93.9%, the cumulative success rates are of interest. The success rate after 10 years was 98.9%, after 15 years 96.7%, after 20 years 93.9%, after 25 years 87.5% and after 30 years 78.5% for hnPCCs. In the present study, 92.7% of the patients were clinically monitored for more than 10 years in the study practice.

## Discussion

According to the results of this investigation, hnPCCs continue their eligibility as an option for restorations of decayed teeth in dentistry with excellent long-term prognosis supporting the working hypothesis. In the present retrospective study, the survival and success rates of 1325 hnPCCs were evaluated. A survival rate of 94.9% and a success rate of 93.9% were determined after a mean observation period of 18.8 ± 5.7 years. Thereby, 92.7% of the examined hnPCCs showed regular follow-ups for more than 10 years. Even the hnPCCs that had to be documented as unsuccessful showed a mean of 16.2 ± 6.2 years of service. This comprehensive investigation was possible due to the fact that in Germany, the loyalty of a patient to his practitioner is specified with approx. 90% [32]. The high number of reexamined restorations in combination with a very extensive observation period allows for a more accurate prognosis regarding the success and survival probabilities of partial-coverage crowns manufactured from high-noble metal alloys.

With reference to existing literature, a reliable statement regarding long-term success rates of hnPCCs is difficult, since most studies did not exceed 10 years of follow-up data (Table 3). Additionally, the study designs were discussable regarding its number of investigated hnPCCs and materials [8, 25]. Compared to other studies, a success rate of 93.9% after a mean observation period of 18.8 ± 5.7 years is very high (Table 3). This might be explained by the highly specialized and experienced restorative team and the matured treatment concept. In comparison, other studies (Table 3) documented various dentists, dental technicians, materials and preparation designs [19]. Thereby, a regular recall of patients was not present [19, 33]. However, the fact that only patients with regular follow-ups and/or cleanings every 6 months were included in this study demonstrates the high compliance. This might be considered as positive influence on both survival and success rates documented and should also be discussed as bias.

**Table 3:** Comparative studies on survival rates of metallic dental restorations

Authors/Publications	Number of investigated restorations	Mean observation period (in years)	Success rates (in %) and absolute numbers
Creugers et al. 1990 [34]	203	5.0	62% (126)
Creugers et al. 1992 [35]	203	7.5	75% (153)
barrack and bretz et al. 1993 [36]	127	5.8	93% (118)
Samama et al. 1996 [37]	145	5.7	83% (134)
Studer et al. 2000 [38]	303	18.7	86% (261)
Aggstaller et al. 2008 [39]	84	6.3	77% (72)
Opdam et al. 2011 [40]	22	7.0	68% (18)
Botelho et al. 2014 [41]	211	9.4	84% (278)
King et al. 2015 [44]	771	13.0	81% (619)
Tanoue et al. 2016 [19]	311	13.9	73% (327)
Najafi et al. 2016 [45]	198	8.5	69% (137)
This study	1325	18.8	93.9% (1244)

Further attention should be paid to the used alloy and its material characteristics. In this study, Degulor M (a high noble metal alloy) was used for the manufacturing of all hnPCCs. The ductility of gold alloys seems to have a decisive influence regarding the probability of success. It is assumed that restorations made of high-gold alloys correspond to the degree of hardness of its antagonists and thus ensure occlusal stress reduction [18]. Due to the property of gold to permanently deform plastically under shear stress prior to fracture, stress fractures as those found in ceramic restorations are not existent. Even with possible wear of adjusted front-canine guides, it is accepted that the ductility of gold leads to fewer hyperbalances in contrast to ceramic occlusal surfaces [16]. Likewise, it is assumed that a restoration with cPCCs in the posterior region is protected by an intact front-canine guide. In the case of natural abrasion of this guide over decades, this protection is no longer available [18].

Furthermore, success of hnPCCs is dependent on its preparation and restoration design. Special attention was paid to the preparation design and to maintain as much tooth structure as possible. Bevelled preparation edges were used to minimize cement gaps [23]. Covering all cusps and thus the entire occlusal surface appears to increase the stability of the restoration and residual tooth substance is less affected by prismatic fractures [19, 24] (Fig. 1e). Comparing hnPCCs with cPCCs, ceramic materials and adhesive bonding have improved in the last decades [42, 43]. Today, similar results to metal restorations can be achieved with cPCCs [34–36]. However, in clinical studies, chipping of veneering ceramics has been shown to be the most frequent reason for failure [6–8, 34–37]. It was also described that bruxism and the missing involvement of cusps negatively influence the survival rate of cPCCs compared to hnPCCs [38–40]. In a meta-analysis, Morimoto et al. [36] examined 14 studies on ceramic restorations from the period between 1983 and 2014; all of which were in vivo studies with non-preselected patients who participated in regular recalls for at least 5 years. The collected results in this meta-analysis described a success rate of 95% after 5 years and 91% after 10 years. It was assumed that the adhesive bonding was a decisive factor regarding the success of the ceramic restoration [36]. Although the documented success rates for cPCCs should be rated as very high, the investigated success rates of hnPCCs in this study are even higher with 93.3% and with a mean observation period of 18.8 years.

The main reason for a rating as an unsuccessful restoration (hnPCC) were periodontal issues. This study documented that success rates became significantly worse after a mean age of 51 years comparing the different age groups with each other (*p* = 0.012) (Table 2). Periodontal diseases increase with age [41] and cause tooth loss due to loss of attachment, which in turn leads to loss of the respective restoration. Higher age as a decisive factor for success could be due to the fact that patients in the upper quartile are no longer able to maintain adequate oral hygiene due to motor limitations. In contrast, a plausible explanation for the lower success rates in patients under 37 years of age is difficult. An assumption might be that young patients receiving dental restorations already this early suffer from lack of compliance and dental hygiene.

The second most frequent complication were decementations of hnPCCs. Debonding of cPCCs occurred in 1% of the restorations (*n* = 24 of 4.854 cPCCs) and is therefore comparable with the documented decementation of 1.1% hnPCCs in this study but within a shorter observation period [36].

A main issue regarding the comparability of ceramic and metallic partial dental crowns are the various observation periods of the studies, especially for cPCCs. The success rate of cPCCs is documented with 95% after 5 years [36] and therefore comparable or even better than results for hnPCCs (Table 3), but within a shorter observation period. In the meta-analysis of Morimoto et al. on cPCCs, there were only three studies with a monitoring period of 15 years. The mentioned success rates were 81.5% (Beier et al., 2012), 88.7% (Otto and Schneider, 2008) and 89% (Reiss, 2006) [36]. Also, with regard to hnPCCs, few studies with longer observation periods than 10 years are available. Precisely, the very long observation period and number of cases are decisive strengths of the present study (Table 3). The results are therefore transferable to everyday clinical practice and present a strong statement, with the restriction that the treating clinician embodies high clinical standards and sticks to the treatment concepts. However, this should be the claim of every practitioner. While it can be stated that cPCCs show comparable success rates to hnPCCs in the first 5 years, the long-term prognoses of cPCCs are not yet clearly clarified.

A retrospective investigation depends on the selection and documentation of the available data. For the individual cases, unfortunately, not as much data as desired were documented in the records and hence could not be analyzed. Furthermore, due to the design of this study, it was not possible to subsequently change study criteria, evaluate different treatment concepts or include a control group. However, only studies with reproducibility, high number of cases and a longest possible observation period can provide information on the long-term prognoses of treatment concepts. All steps from diagnosis to restoration were performed by a single dentist and a single lab technician in a privately run dental practice. Over the entire treatment period, the used alloy and materials were the same and the workflow was standardized. This should of course also be critically discussed as bias, i.e. regarding objectivity towards the own work. Inclusion of an external reviewer would have been desirable to present more objective results with better transferability. Thereby, it should be noted that the results were worse in studies with various practitioners [19, 26].

## Conclusion

Partial-coverage crowns fabricated from a high noble metal alloy exhibited an excellent clinical long-term performance with a survival rate of 94.9% and a success rate of 93.9% after a clinical service time of up to 30 years. The documented treatment concept is still entitled to be considered in modern restorative dentistry.
